# Electrostatics of Tau Protein by Molecular Dynamics

**DOI:** 10.3390/biom9030116

**Published:** 2019-03-23

**Authors:** Tarsila G. Castro, Florentina-Daniela Munteanu, Artur Cavaco-Paulo

**Affiliations:** 1 Aurel Vlaicu University of Arad, Str. Elena Drăgoi 2-4, RO-310330 Arad, Romania; castro.tarsila@ceb.uminho.pt (T.G.C.); florentina.munteanu@uav.ro (F.-D.M.); 2 Centre of Biological Engineering, University of Minho, Campus de Gualtar, 4710-057, Braga, Portugal

**Keywords:** tau, microtubules, electrostatics, diffusion, protein structure prediction, molecular modelling, molecular dynamics, tau–microtubule association

## Abstract

Tau is a microtubule-associated protein that promotes microtubule assembly and stability. This protein is implicated in several neurodegenerative diseases, including Alzheimer’s. To date, the three-dimensional (3D) structure of tau has not been fully solved, experimentally. Even the most recent information is sometimes controversial in regard to how this protein folds, interacts, and behaves. Predicting the tau structure and its profile sheds light on the knowledge about its properties and biological function, such as the binding to microtubules (MT) and, for instance, the effect on ionic conductivity. Our findings on the tau structure suggest a disordered protein, with discrete portions of well-defined secondary structure, mostly at the microtubule binding region. In addition, the first molecular dynamics simulation of full-length tau along with an MT section was performed, unveiling tau structure when associated with MT and interaction sites. Electrostatics and conductivity were also examined to understand how tau affects the ions in the intracellular fluid environment. Our results bring a new insight into tau and tubulin MT proteins, their characteristics, and the structure–function relationship.

## 1. Introduction

**Figure 1:**
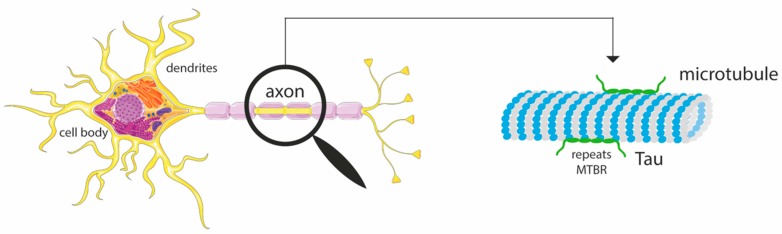
Graphical representation of a neuron, highlighting a microtubule present in the axon and the interaction with the tau protein, through the four repeats at the microtubule binding region (MTBR). Adapted from Servier Medical Art [5].

Tau is an intrinsically disordered protein (IDP) that stabilizes and promotes the assembly of microtubules (MTs) in neurons (Figure 1); it is, therefore, a microtubule-associated protein (MAP) [1]. Tau is involved in cell polarity and, as it is distributed along the axons, is responsible for the maintenance of neuron structure and healthy function [2,3]. Structurally, tau is also considered as a spacer between adjacent MTs, although that is not its main function [4].

**Figure 2:**
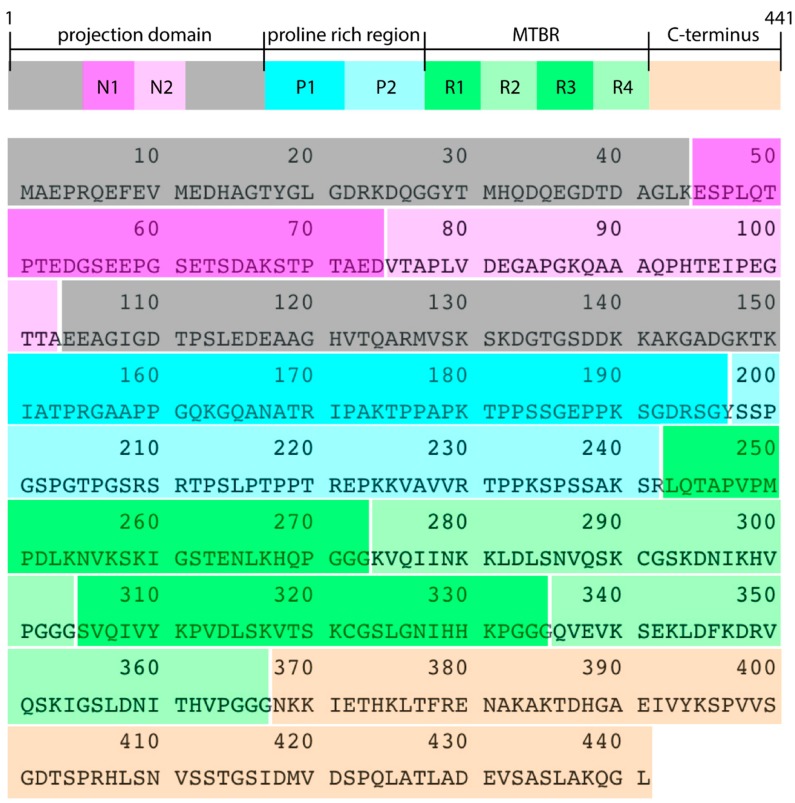
Primary sequence and regions of 2N4RTau isoform, comprising 441 amino acids. N1 and N2 correspond to the two N terminal inserts, P1 and P2 are the proline-rich regions, and R1 to R4 are the four imperfect repeats responsible for the binding to the microtubule.

The adult largest tau isoform has 441 amino acids, comprising a projection domain with two amino-terminal inserts N, encoded by exons 2 and 3, a proline-rich region and the microtubule binding region (MTBR), with four imperfect repeats (Figure 2). This isoform is commonly cited as 2N4RTau, isoform-F, htau40 and Tau-4, and is the largest one in the human central nervous system (CNS), which has five other isoforms [6]. 

Besides the relevant structural support function, that comes from the interaction between tau and the tubulin MTs, tau plays an important role in nervous system diseases. If hyperphosphorylated, tau does not perform its normal function, but aggregates in paired helical filaments (PHF) [7,8] which inhibits the MT assembly. The PHFs cause tau lesions found in Alzheimer’s disease (AD) brains and in other types of dementia [9,10,11,12].

Several post-translational modifications can occur in tau, namely, phosphorylation, glycosylation, nitration, glycation, ubiquitination, among others [13,14]. The degree of these modifications is what will lead to a normal or pathological functioning. In particular, tau phosphorylation is the most studied alteration, as it is established that the abnormal hyperphosphorylation is associated with AD [15,16], a dementia that currently has no cure. 

To date, tau’s complete structure has not been fully solved experimentally. Only portions of the canonical sequence are known, or hyperphosphorylated sections, as listed in UniProt (Universal Protein resource) [17] and in the RCSB PDB (Research Collaboratory for Structural Bioinformatics Protein Data Bank) [18]. This fact hinders a more complete understanding of the structure–function relationship for this protein. Indeed, the information concerning tau structure bound to MT is controversial [19,20,21], even in the most recent papers on the subject [22,23].

Our work sheds light on the tau three-dimensional (3D) structure. We performed the complete modelling of the 2N4RTau isoform, using protein structure prediction (PSP) techniques, and submitted the obtained model to molecular dynamics (MD) simulations in order to characterize the following: tau profile in neuron fluid, its interactions and structure when associated with MT, the effect of tau on ions’ diffusion and conductivity, among other properties. 

## 2. Materials and Methods

### 2.1. Tau Structure Prediction

Tau conformation was predicted using the I-TASSER (Iterative Threading ASSEmbly Refinement) server, a method to predict protein structure and function that uses a multiple threading approach based on templates from the Protein Data Bank (PDB) [24]. The I-TASSER is consistently considered the best server for PSP in community-wide CASP (Critical Assessment of protein Structure Prediction) experiments [25,26]. The threading templates used to predict tau structure were the proteins with the following codes: 2nbi, 3zue, 2mz7, 1w0r, 4dur, and 1ziw. 2nbi corresponds to pleuralin-1, a structural protein that shares 19% of sequence identity with tau. 3zue is a virus capsid protein presenting 22% of overall sequence identity with tau. 2mz7 is the only template that matches an NMR tau structure (amino acids 267–312) [21], and fulfil 10% of similarity. 1w0r is properdin, a glycoprotein with 20% of sequence alignment with tau. 4dur corresponds to the X-ray structure of type II human plasminogen, a hydrolase that shares 17% of similarity with tau, and 1ziw is the human toll-like receptor 3, making a total of 17% of identity with tau. 

The I-TASSER server starts the modelling from the structure templates mentioned above, identified with LOMETS (Local Meta-Threading-Server), but other procedures and programs take place to generate the final five hit models. From this list we chose the one with the best C-score. The model was predicted in 2017 with the template structures available that year. 

### 2.2. System Setup

**Figure 3:**
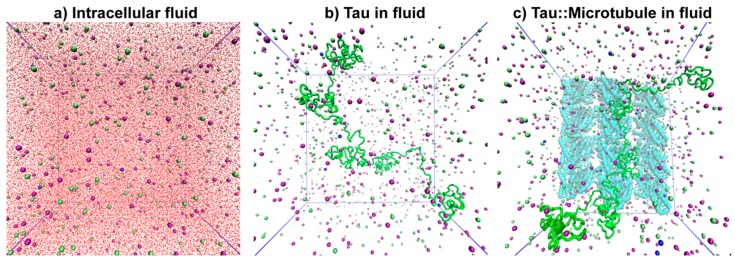
Front view snapshots of the systems under simulation: (**a**) Intracellular fluid, (**b**) tau in fluid, and (**c**) tau::microtubule wall in fluid. Water molecules were omitted in (**b**) and (**c**) to allow better visualization. Tau is represented in green, microtubule in cyan, K^+^ in purple, Na^+^ in blue, and Cl^−^ in green spheres.

Three types of systems were designed to study tau (Figure 3). The first one was a simulation box composed only of neuronal intracellular fluid as control (Figure 3a). The second was a simulation box containing the predicted tau structure in the neuronal fluid (Figure 3b), and the third contained tau next to a tubulin wall (an MT section (PDB ID: 5JCO)) in the fluid (Figure 3c). The second and third systems are hereinafter referred as tau in fluid and Tau::MT in fluid. For tau in the fluid, the protein was centered in the box, but in the case of Tau::MT systems, the tubulin wall was placed at one of the walls of the box and tau randomly near to MT, as shown in Figure 3.

The fluid inside the neurons was composed of water, a high concentration of potassium and proteins with negative character, and a discrete concentration of sodium and chloride among other ions [27]. In the simulations performed, we fixed the concentration of K^+^ and Na^+^ to the reported values [27,28,29]: 140 mM for K^+^ and 5–15 mM for Na^+^ at the neuron resting state. These cations are the ions involved in the transmission of electrical signals in these cells and, most importantly, the probability of being affected by the negatively charged MT wall and by tau is high.

As it was not feasible to simulate explicitly all cytoplasmic proteins in neuronal fluid, since it would require a greater computational power, we decided to replace the negative contribution from these proteins for Cl^−^. The final balance of charges, in the simulation boxes, would be the same using negatively charged proteins or Cl^−^ ions. In addition, as described below, we used particle mesh Ewald (PME) for electrostatic treatment and periodic boundary conditions (PBC) in our simulations, therefore the simulation only formally converges if the net electric charge is zero. That is why the addition of ions to achieve neutrality is a common procedure in MD simulations.

In all three cases, a simulation box with an approximate volume of 7200 nm^3^ was necessary. The final number of simple point-charge (SPC) water molecules depended on the volume occupied by tau or the pair Tau::MT.

### 2.3. Molecular Dynamics Simulations

Molecular dynamics simulations were performed on the systems described above, to understand tau structure and properties. 

Two stages of energy minimization were performed using a maximum of 50,000 steps: the first using the steepest descent method and the second with the conjugate gradient algorithm [30]. Initialization steps using canonical (NVT, constant number of particles, volume and temperature) and isothermal-isobaric (NPT, constant number of particles, pressure and temperature) ensembles were performed applying position restraints (with force constant of 1000 kJ·mol^−1^·nm^−2^) to all heavy atoms in the NVT procedure, and to the main chain at the NPT initialization step, during 100 ps each. In the control situation (fluid), no position restraints were applied. The temperature was maintained constant at 310 K with V-rescale algorithm [31] and the pressure was regulated at 1 atm with the Parrinello–Rahman barostat [32]. The following coupling constants were considered: τ_T_ = 0.10 ps and τ_P_ = 2.0 ps. Subsequently, all systems were submitted to MD simulations: the fluid during 10 ns, tau in fluid during 60 ns, and Tau::MT in fluid for 70 ns, all using the NPT ensemble, without position restraints. Three replicates of each system were run to guarantee a better sampling of conformation states for these very large proteins under study.

All MD simulations were performed using the computational package GROMACS 5.1.4 version [30,33], within the GROMOS 54a7 force field (FF) [34,35]. The Lennard-Jones interactions were truncated at 1.4 nm and we used the particle mesh Ewald (PME) [36] method for electrostatic interactions, with a cut-off of 1.4 nm. The algorithm LINCS [37] was used to constrain the chemical bonds of the proteins and the algorithm SETTLE [38] in the case of water. Parameters for K^+^ in the scope of G54a7 FF were obtained from the paper of Cordomí et al. [39].

### 2.4. Analysis

From the MD simulations, we analyzed the trajectories looking at the root-mean-square deviation (RMSD) to determine when the systems were at equilibrium. Based on the RMSD results, the following analyses were performed from 30 ns.

Root-mean-square fluctuation (RMSF) analysis and CLUSTER analysis with the single-linkage method were used to understand regions and amino acids with greater flexibility and to determine the middle structure of each tau replicate, respectively. This technique adds structures that are below an RMSD cut-off, generating more or less populated clusters and, within the largest cluster, it finds a middle structure that is the most representative of the whole simulation. Radial distribution functions (RDF) were calculated for the positive ions around the tau surface.

GROMACS’ mean square displacement (MSD) was used to calculate the ions’ mean square displacement in all systems. To calculate the diffusion coefficient (D*_i_*) of the particles, one can use the Einstein relation [40], illustrated in Equation 1, where *r_i_* corresponds to the particle center of mass position at a certain time *t*:

(1) $$D_{i}=\underset{t\to \infty }{\lim}\frac{1}{6t}\;\langle ∥r_{i}\left(t\right)-r_{i}\left(0\right){∥}^{2}\rangle .$$

From the D*_i_* obtained with this analysis, it is possible to calculate the molar conductivity, as demonstrated in Equation 2. The Nernst–Einstein equation (2) establishes the relationship between the molar limiting conductivity Λ*_m,i_* and the diffusion coefficient D*i* for any given ion *i*, at a certain temperature (T). In the equation, *z* corresponds to the charge of the ion *i*, *F* corresponds to Faraday’s constant, and *R* is the gas constant. Here, our conductivity calculation is an approximation, since our systems do not represent an infinite dilution of the ions (non-interacting ions). We disregarded the ion–ion correction factor to use Equation (2) directly:

(2) $$\Lambda_{m,i}^{{}^{\circ}}=z_{i}^{2}D_{i}\left(\frac{F^{2}}{RT}\right).$$

The MSD was calculated for the total simulation time but considering time intervals of about 10 ns. We chose to do this because this analysis requires a large memory capacity for large systems and to ensure reliable MSD data, resulting in a linear MSD(t) plot for each “-b to -e” time interval option. All analysis programs used are available in the GROMACS 5.1.4 package [29,32].

The analyses of the electrostatic potential of the predicted tau structure and the microtubule section (5JCO) were performed using the PDB2PQR server [41] and the APBS (Adaptive Poisson-Boltzmann Solver) plugin in PyMOL molecular visualization program [42,43,44]. For the microtubule we calculated the electrostatic surface for a tubulin heterodimer, since it is representative of the whole structure.

All figures presenting molecular structures were made with PyMOL and VMD (Visual Molecular Dynamics) software [45].

## 3. Results and Discussion

### 3.1. Tau Structure Prediction and Validation

**Figure 4:**
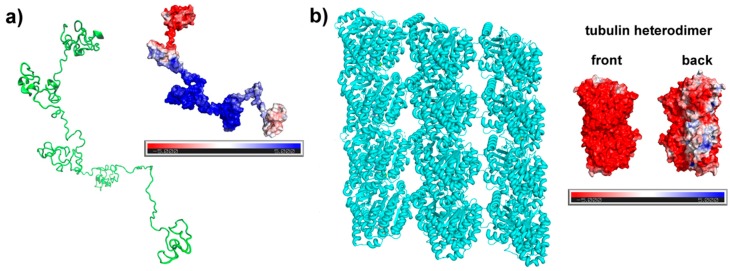
(**a**) Predicted structure of tau using I-TASSER and (**b**) 5JCO microtubule section from Protein Data Bank (PDB). Both structures are side by side to its electrostatic potential surface, which in red represents the negative potential values and in blue, the positive potential values. The electrostatic potential (k_b_ T e_c_^−1^) was calculated using APBS-PDB2PQR software. For the microtubule, the calculation was made using a tubulin heterodimer.

Tau conformation was predicted with the I-TASSER server (Figure 4a). This method generates five hit models, from which we chose the one with the best C-score. The predicted tau is an elongated structure with only two small portions presenting a well-defined secondary structure (SS). The lack of SS is consistent with what is cited in the literature, which describes tau as an intrinsically disordered protein [46,47]. This extended form is important to the tau function, as it allows a proper exposure, flexibility, and contact of the MTBR residues with tubulin MT [48].

Some authors describe how the MTBR can gain some helical SS when interactions take place with MTs or actin filaments [19,49]. Recently, Zabik et al. [23] performed NMR studies in tau peptide fragments and in full-length tau, but tau easily forms oligomers, influencing tau 3D structure. Moreover, tau in solution adopts a more compact conformation, with N- and C- termini interacting and inducing a paperclip structure (i.e., a folding over the MTBR that approximates the terminals). This conformation contrasts with the extended conformation required for interaction with MT but is the most typical in solution [23,50,51,52]. To address both situations, tau in intracellular solution and tau bound to MTs, we simulated these two systems to disclose the tau structure in different contexts.

Predicted tau presents an electrostatic surface highly positive in the central region and a negatively charged patch at the N-terminal. Tau electrostatics reinforces the approximation between terminals that occurs in solution, folding over the middle domain of tau and resulting in a paperclip conformation.

In the case of tubulin heterodimer, the constituents of a microtubule, the surface is predominantly negative with few neutral or positive patches on the back (microtubule interior). The electrostatic surface that we calculated is very similar to the one presented by Baker et al. for a larger microtubule structure [43].

**Figure 5:**
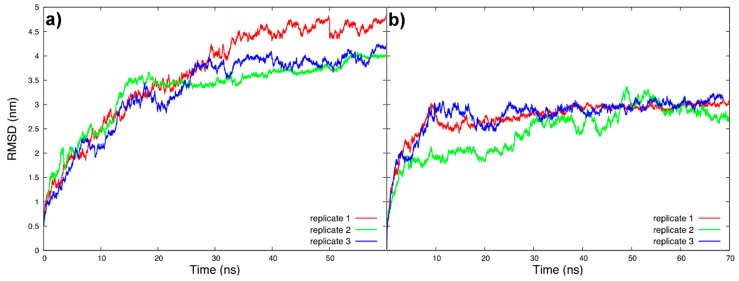
Backbone root-mean-square deviation (RMSD) of tau, fitting the backbone, for all tau replicates (**a**) in fluid and (**b**) when associated with microtubule, from the initial predicted model.

Root-mean-square deviation is the most used analysis to monitor the structural behavior of a protein—either to see the maintenance of a tertiary structure (protein stability in a medium) or to follow the equilibration of a sampled conformation (the folding). In the case of the tau protein, we started from a model structure that needed to be equilibrated in its most typical environment, that is, embedded in the neuronal fluid, interacting or not with MTs. A large structural deviation was expected at the beginning of the simulation as well as a conformational variety. Our systems, tau in fluid and Tau::MT in fluid, took about 30 ns to reach equilibration (Figure 5a,b) and to start sampling conformations more similar among each other.

**Figure 6:**
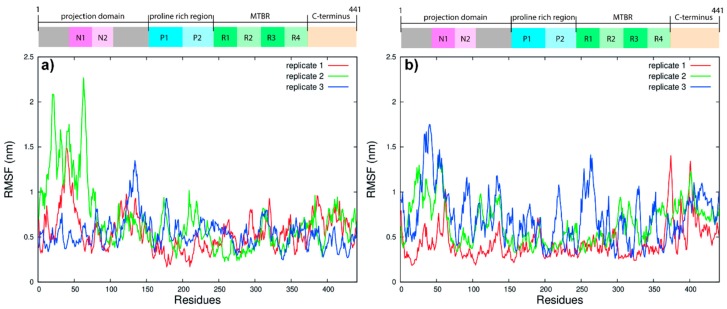
Residue root-mean-square fluctuation (RMSF) (**a**) of each tau replicate in fluid and (**b**) of tau::microtubule pair in fluid.

The RMSF provides an insight into tau residue mobility, indicating the more rigid and more flexible regions. In fluid (Figure 6a), tau is more flexible at the N-termini region and projection domain. From the proline-rich region, it becomes less fluctuating. When near to the MT wall (Figure 6b), the amount of interactions between tau and MT (electrostatics, van der Waals and hydrogen bonds), which must form and break repeatedly, induces a more pronounced fluctuation throughout the tau structure, with proline-rich region and MTBR being the most rigid regions for replicates 1 and 2. This fact is in agreement with the strongest interaction of MTBR with the MT wall, reducing the mobility of this area.

**Figure 7:**
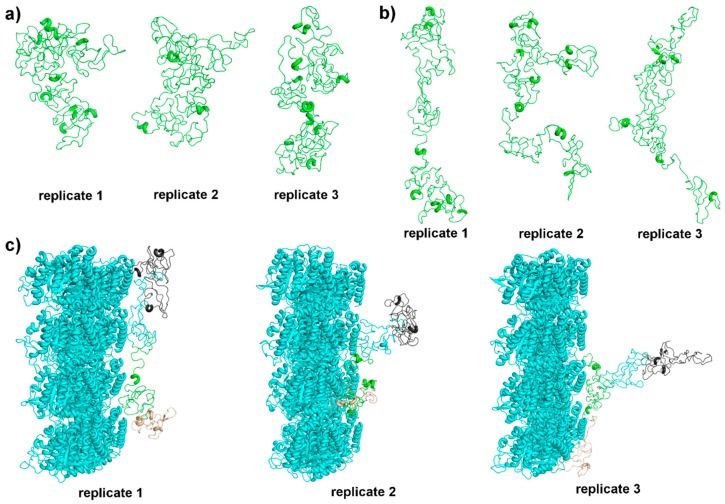
(**a**) Middle structures of tau in fluid, (**b**) middle structures of tau when associated with microtubules, with N-termini oriented to the top, and (**c**) tau::microtubule middle structures, with a color scheme following Figure 2: grey for N-terminal, cyan for proline-rich domain, green representing the MTBR, and beige for C-terminal. (**c**) Microtubule wall is represented in cyan cartoon and (**a,b**) tau is represented in green cartoon.

The cluster analyses were employed from 30 to 60/70 ns to determine the middle conformation of each replicate (Figure 7a,b). Middle structures for tau in fluid also have helical SS in MTBR, namely, replicate 1: amino acids 275–277 and 315-318; replicate 2: amino acids 315–319; and replicate 3: amino acids 253–259, 269–273, 280–282, and 359–362. However, these structures seem to fold over themselves, becoming more compact and less exposed, agreeing with tau NMR prediction in solution [23]. Visually, the structures obtained for Tau::MT in fluid are more similar and extended. In fact, all three present helical content in MTBR, namely, replicate 1: amino acids 274–277 and 305–310; replicate 2: amino acids 256–260 and 357–362; and replicate 3: amino acids 253–257 and 350–354. In addition, for tau from Tau::MT replicates, we observed a more extended structure with N- and C-termini far from each other.

To better understand the interaction between tau and the tubulin MT wall, the middle conformations for the pair Tau::MT were also calculated. Figure 7c shows these pairs highlighting the N-terminal, proline-rich region, MTBR, and C-terminal with the color scheme proposed in Figure 2.

Replicates 2 and 3 interact with parts of two tubulin heterodimers and the N-termini are further apart from the tubulin wall than in the case of replicate 1. There is no consensus on the type of tubulin (α or β) to which tau binds preferentially, on the number of units [48,53], or even if the interaction is longitudinal or lateral [54]. More recently, Kellogg et al. [22] proposed an atomic model of tau bound to MT, suggesting that each tau repeat spans over three tubulin monomers as a continuous stretch, even more extended than our middle structures and reaching a higher number of tubulin monomers. The determination of the interaction mode between tau and MT has to take into account the highly dynamic behavior of tau and its ability to disconnect from one MT to connect to another in the neighborhood, in a kiss-and-hop mechanism, as cited by Janning et al. [55], suggesting that tau binding might be slightly different in interactions each time.

Electrostatic interactions and hydrogen bonds take place in all three replicates associated with MT, although for replicate 3, only the MTBR (Thr263, Lys281, and Lys290) and C-termini interact. Tau replicate 1 interacts along all its structure, with important interactions at the proline-rich region and MTBR (Ser202, Lys224, Arg230, Ser237, Ser241, Val306, Ser316, and Lys317). Replicate 2 interacts a little less with the proline-rich region (Ser202, Val228, and Lys240), much with the MTBR (Arg242, Lys274, Lys 280, His268, Gln288, Cys291, Gly308, and Leu315) and through the C-termini. It has to be stressed that the amino acids cited above are mostly polar and many of them positively charged, resulting in a good binding to the acidic C-terminal of tubulins (cyan helices, near tau, in Figure 7c).

Structurally, tau 3D structure, when tau interacts with MTs, is more extended than tau in solution, with the termini far from each other. If oriented to the same direction, all three replicates are quite similar (Figure 7b), which is a very good result for a simulation of a very dynamic IDP, under the interaction forces from the MT wall. In addition, we observed that tau replicates sample helical portions at the MTBR, as predicted or suggested in some works [19,46]. It is important to note that at the end of each MTBR repeat, there is a PGGG motif. Proline is a helical breaker and glycine, due to the absence of a side chain, is highly flexible and rarely found in helices. This motif should correspond to an extended or bended region that will connect the four repeats. In fact, in our simulations, PGGG does not sample secondary structure, and its inherent flexibility causes repeats not to be completely extended along the MT wall, but to approach one another.

### 3.2. Tau Effect on Ionic Diffusion and Conductivity

To analyze the ions’ behavior, the RDF and the diffusion coefficients were studied. This is important to understand the role of tau or Tau::MT pair in the distribution of the ions present in the intracellular fluid. The RDF describes how the density of a particle varies as a function of distance from a reference molecule. Following this rationale, we can perceive how the K^+^ and Na^+^ ions interact with tau and MT.

**Figure 8:**
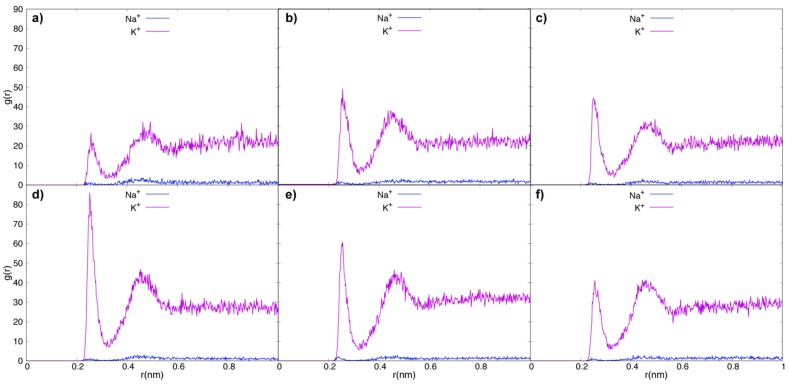
Radial distribution functions (g(r)) of K^+^ and Na^+^ ions in the vicinity of the tau surface, for tau replicates in fluid (**a**–**c**) and tau::microtubule replicates (**d**–**f**). Purple trace for potassium and blue trace for sodium.

Figure 8 shows the RDF graphs for K^+^ and Na^+^ ions for all three replicates of tau in fluid (Figure 8a–c) and Tau::MT in fluid (Figure 8d–f). This analysis helps to understand the role of the electrostatic interaction with the proteins, in the ions’ mobility through the fluid. In all cases, K^+^ has a higher probability of being closer to the tau negative area (N-termini) than Na^+^. Note that the concentration of both ions is very different (interfering with the probability), namely, 140 mM for K^+^ and around 5 mM for Na^+^, yet sodium is lighter than potassium and could be more mobile and interact with tau more often, but an interaction preference with K^+^ is perceived. In this analysis, we focus on the ions’ distribution up to 1 nm from the protein surface. Naturally, the ions also populate the bulk water, corresponding to a high probability far from protein, but we neglected the distribution in this area. If tau N-terminal retains K^+^, it can keep these ions further away from the cell membrane inner face, increasing the membrane potential.

**Figure 9:**
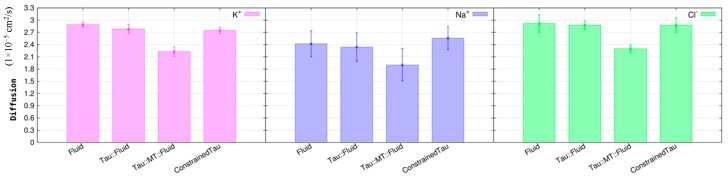
K^+^, Na^+^, and Cl^−^ diffusion, D*_i_* (1 × 10^−5^ cm^2^/s), in the different systems under study: Neuron intracellular fluid (Fluid), tau in fluid (Tau::fluid), tau::microtubule in fluid (Tau::MT::Fluid) and constrained tau in fluid (ConstrainedTau).

The ion’s diffusion coefficients were calculated for K^+^, Na^+^, and Cl^−^ (Figure 9). Chloride is used for two reasons: to mimic the negative contribution from cytoplasmic proteins and to neutralize the simulation box. In fact, Cl^−^ is more mobile than the proteins in neuronal cytoplasm, but tau and MT should be less sensitive to this negative ion, especially MT due to its electrostatics. Therefore, the interaction pattern of Na^+^ and K^+^ with tau and MT proteins is probably little influenced by chloride in our simulations.

Experimentally, the ions’ diffusion and conductivity are always expressed at 25 °C, not at 37 °C. In addition, this calculation is made at infinite dilution, so in our systems we have to consider the concentration effect, which makes a direct comparison to experimental values difficult. However, the trend of Na^+^ < K^+^ < Cl^−^ in terms of diffusion and conductivity is expected in all simulated systems.

In the first simulation, the intracellular fluid, the ions were free to move around the simulation box, respecting the intracellular concentration and in the presence of chloride ions to neutralize the system. We observed that the diffusion (Figure 9) follow the expected trend of Na^+^ < K^+^ < Cl^−^ found in many ionic solutions [56].

Looking at the ions in the system tau in fluid, we emphasize that the presence of tau does not decrease the ions’ diffusion. All three ions maintain the diffusion rate, if we consider the estimated error (Figure 9). In contrast, when tau is directly associated with the MT (Tau::MT replicates), there is a decrease in the diffusion. It was reported that tau diffusion along an MT lattice is influenced by the ionic strength and pH, especially by K^+^ [57]. This suggests that when tau is near or attached to the MT, the ions’ diffusion through the fluid should decrease as the ions are participating in the protein diffusion process.

To circumvent the direct contribution of MT in ionic diffusion, we simulated a tau middle structure (from Tau::MT replicate 2) attached to the box wall, and constraining the MTBR. Thus, we obtained the expected structure when tau interacts with the tubulin wall, but only the tau effect on the conductivity is observed.

**Figure 10:**
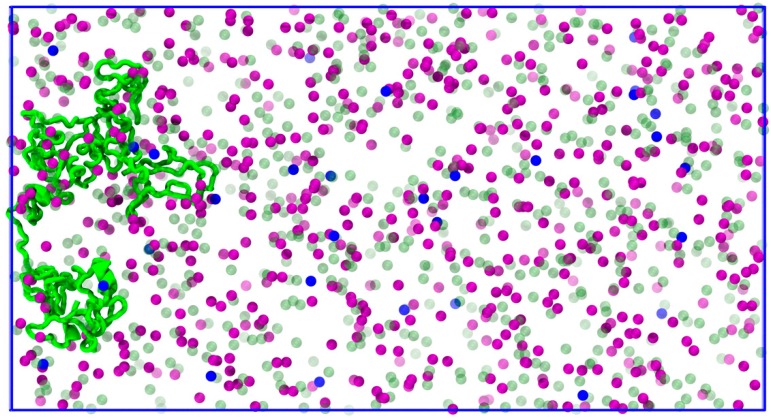
Orthographic view of constrained tau to the box wall. Tau is represented in green cartoon, K^+^ in purple, Na^+^ in blue, and Cl^−^ in green transparent spheres.

A simulation box with the same volume and ion concentrations was modelled during 12 ns (Figure 10). We observed, in this case, that tau increases the diffusion of the Na^+^ cation, possibly making this ion more available in the fluid to move through the sodium channels and across the membrane, as the probability of tau to retain K^+^ is much bigger than to retain Na^+^ (radial distribution functions in Figure 8). The diffusion of K^+^ is once again similar to the one calculated in the intracellular fluid, reinforcing the statement that tau is a protein that does not prevent the normal movement of this ion and the consequent concentration balance in the interior and exterior of the cell.

Predictions for ion membrane diffusion in neurons indicate a D_K_^+^ of 1.96 cm^2^/s and a D_Na_^+^ of 1.33 cm^2^/s [58]. In addition, the potassium diffusive coupling has been reviewed in neural network processes [59], but less information is mentioned for the behavior of the ions in the axonal region, that is, when exclusively embedded in the intracellular medium. Our results for K^+^ and Na^+^ diffusions are slightly higher than the cited literature for transmembrane diffusion, but they result in almost the same difference between these ions.

Looking now to the calculated conductivities (Table 1), we observe very similar conductivity values in all systems, except for Tau::MT in fluid. In general, proteins affect the conductivity in two ways: by carrying a charge and by influencing viscosity. Therefore, it is acceptable that the MT, a bulky and negatively charged protein wall, has the effect of diminishing the global conductivity. The MT has a very negative electrostatic profile that retains the cations, decreasing the diffusion and the conductivity.

Our findings indicate that tau in its dephosphorylated form (i.e., when performing its normal function, with the proper conformation in solution and/or associated with MT) maintains the normal K^+^ diffusion and discreetly increases Na^+^ diffusion, which, in fact, must be more available to leave the inside of the neuron. The biological relevance of this finding is still unclear, but tau may have a role in the electrical signal transmission along the axon.

## 4. Discussion

The present study unveils tau’s structural preferences and electrostatics, when this protein is free in solution (intracellular fluid) or bound to microtubule in neuron fluid. The electrostatic potential surface of tau (Figure 4) reveals a dipolar protein, where the proline-rich region and MTBR are predominantly composed of basic amino acids (positive charge from side-chains). On the other hand, the N-terminal is acidic, with several amino acids negatively charged at physiological pH. Looking at the tubulin MT potential surface, a very negative electrostatic potential can be observed, especially for the exterior (front), indicating that the binding of tau to its wall will occur through the positive region of this protein, and the N-terminal will tend to move away from the wall, as much as possible. In fact, it is widely reported that the binding occurs between tau MTBR and MT, and the electrostatic distribution of both proteins indicates that this is the best choice to prevent repulsion.

The work of Guo et al. [60] (and references therein) summarizes tau’s structural basis and points to a paperclip conformation in solution [50], where the terminals are in close proximity, as we verified with our simulations. Kellogg et al. provided, in their work published last year, a near-atomic model of Tau::MT interactions [22]. In contrast to the paperclip model, observed in solution, they determined that tau is in an extended form, in which each MTBR repeat interacts with one tubulin dimer and with its interface to the next tubulin. This configuration separates the terminals, which agrees with what we see for tau in our Tau::MT simulations (Figure 5), although we sampled a less elongated MTBR. Under dynamics, the PGGG motif localized in each repeat causes consecutives bends that result in a folded MTBR, thus our simulated model interacts with a small number of tubulin monomers. Summing up, structurally, when we simulate our tau model, it behaves as expected in solution, as a paperclip, and shares similarities with the findings of Kellogg, such as the distance between terminals.

Tau performs other important functions in the intracellular neuron environment, besides the well-known functions in MT binding and stabilization [60]. Interestingly, with the exception of the C-termini, each tau domain can be related with the interaction to other proteins. The N-terminal has been involved in the inhibition of axoplasmic transport through a signaling cascade [61] and in interactions with proteins at the neuron plasma membrane [62]. The proline-rich region also interacts with several proteins, specially the Src family of protein kinases [63]. The MTBR has been also associated with an interaction with the lipid membranes [64].

The tau localization in neurons is also an important factor for tau interaction and function. Tau is mainly located in axons [63] and in a much smaller amount in somatodendritic compartments [65,66,67]. Guo et al. [60] summarizes the presence of tau in different neuron locations, but we highlight here the association with neuronal membranes, which is required for tau participation in intracellular signaling pathways [68,69]. It is also in the membrane that the propagation of electrical impulse occurs, through the flow of positively charged ions. The action potential is maintained due to a concentration difference of certain ions. Potassium has a higher concentration inside the cell, and sodium has a higher concentration on the outside. The flux of these ions, using sodium and potassium channels at the membrane, guarantees the process of polarization/depolarization and maintains an ionic balance. Our simulations used concentrations of Na^+^ and K^+^ typical for the resting state.

Tau has been reported as a protein that interacts with many others and also with the cell membrane, inside the neuron [47,60,63]. However, little information is provided concerning the effect of this protein on sodium and potassium ions [57,70]. Our simulations point to a possible novel function or role: due to its polar profile, tau may also play a role in the normal flow of the ions present in the intracellular fluid, as it is highly localized in the axon. The axon propagates the signal, like a wire, and this occurs due to the potential difference maintained by the flow of ions in the axon membrane [71].

Recently, the behavior of tau near a membrane was evaluated. Patel et al. [72] thought that once tau aggregates in a similar way to amyloid peptides, it would be valid to look at its behavior when close to the cell membrane. A tendency for the formation of ion channels, which allow the passage of nonspecific ions, was observed. This fact reinforces the role of tau in the balance of K^+^ and Na^+^ ions within the neuron and in the electrical signal propagation between cells. Our work paves the way to disclose these interactions.

K^+^ and Na^+^ imbalances in neurons were observed in Alzheimer’s disease brains [70]. This fact could arise from the malfunctioning of several proteins, including tau, which causes changes at the structural and signaling level, affecting the normal concentrations of these ions.

Globally, tau has a basic character with a higher content of positively charged amino acids, but among the 27% of charged residues, there are also negatively charged amino acids, which results in a predominantly negative area, the N-terminal (Figure 4). Tau is, therefore, able to interact with ions in an attractive or repulsive manner. In particular, the acidic N-terminal is prone to attracting cations, since it is the tau acidic area and the portion that will interact less with MT, also negatively charged. In fact, RDF and diffusion analyses have suggested that tau retains K^+^ near its surface, but maintaining a normal diffusion, and increases Na^+^ mobility, which is less probable to be near tau.

## 5. Conclusions

Tau modelling and simulations disclose many properties concerning tau structure and function. First, the prediction of a tau 3D structure leads to a model that was equilibrated in solution—intracellular ionic fluid—and associated with an MT wall. In both cases, the secondary structure features observed for the middle conformations are in agreement with experimental estimates: tau in fluid is more compact, with N^−^ and C^−^ termini closer to each other, and with some helical SS at MTBR. Tau in Tau::MT systems is in an extended conformation, with helical portions at the MTBR and with the terminals far from each other.

In terms of interactions, tau interacts with two tubulin heterodimers on average, through hydrogen bonds and electrostatic interactions, mostly established between the basic region of tau and the acidic C-termini of tubulins. Kellogg et al. [22] suggested a more elongated MTBR able to interact with a higher number of tubulin dimers. However, we observed a less extended tau MTBR, due to the great dynamics of this protein.

Concerning the effect of tau on ions, we observed a preference for interacting with K^+^ cations, through the negatively charged N-terminal. More importantly, tau maintains the normal ion diffusion, being a key factor to cell conductivity and signaling. Tau also increases the conductivity of Na^+^ ions, in comparison with the fluid control, in the surrounding environment.

The maintenance of a normal tau structure–function relationship results in a normal interaction with the ions, keeping K^+^ close to the tau surface and Na^+^ ions more available to leave the cell, as expected, to maintain the intracellular concentration balance of both ions at the axon resting state. Our findings about diffusion and conductivity are a starting point to understand, at the molecular level, the participation of tau in the electrical signal propagation across healthy neurons.

In future work, we intend to study different degrees of phosphorylation in our tau model, to evaluate what it will entail in the structure, in the interaction with MTs, and in the ions’ diffusion.

## Figures and Tables

**Table 1:** Calculated conductivity (Λ_i_) at 310 K, for each ion type in fluid, in all systems.

System	Ion	Average Conductivity (Λ_i_) (S·cm^2^/mol)
Intracellular fluid	K^+^	104.21 ± 2.351
	Na^+^	87.26 ± 11.621
	Cl^−^	105.35 ± 7.728
Tau in fluid	K^+^	100.53 ± 3.904
	Na^+^	84.60 ± 12.674
	Cl^−^	103.87 ± 3.667
Tau::MT in fluid	K^+^	80.39 ± 4.117
	Na^+^	68.67 ± 14.327
	Cl^−^	82.93 ± 3.398
Constrained Tau in fluid	K^+^	99.24 ± 2.914
	Na^+^	92.40 ± 10.121
	Cl^−^	103.88 ± 6.523
